# Challenges of vascular access in a new dialysis centre – Uyo experience

**Published:** 2010-12-27

**Authors:** Eyo Effiong Ekpe, Udeme Ekirikpo

**Affiliations:** 1 Cardiovascular and Thoracic Surgery Unit, Department of Surgery, University of Uyo Teaching Hospital, P.M.B. 1136 Uyo, Akwa Ibom State, Nigeria; 2 Nephrology Unit, Department of Internal Medicine, University of Uyo Teaching Hospital, P.M.B. 1136, Uyo Akwa Ibom State, Nigeria

**Keywords:** Vascular access, haemodialysis, challenges, Nigeria

## Abstract

**Background:**

Introduction of dialysis has prolonged the lives of end-stage renal disease patients. To maintain these patients on long term dialysis, permanent vascular access procedures capable of allowing flow of >200ml of blood/minute, are required. Without permanent vascular access, patients are subjected to repeated attempts for cannulation to provide temporary vascular access during every session of haemodialysis, risked with numerous vascular access related complications. The objective of the study was to analyse the problems of vascular access in our new dialysis centre and plan intervention.

**Methods:**

Case notes and dialysis records of consecutive patients who underwent haemodialysis in our dialysis centre during its first one year were used to collect data into proforma, and these were analysed.

**Results:**

There were 60 patients who underwent a total of 254 sessions of haemodialysis during the period. Their ages ranged from 12-72 years. There were 38 males and 22 females. There were 57 patients with end-stage renal disease and three with acute renal failure. Only 5% of the patients underwent dialysis through a permanent vascular access representing 8% of dialysis. The remaining 95% of patients undergoing 92% of haemodialysis utilised temporary vascular accesses. Complications arising from vascular access were noted in 24.0% of dialysis and these included failed or difficult cannulation, poor flow, haematoma, haemorrhage, kinked catheter, thrombosis and infection.

**Conclusion:**

The ratio of temporary to permanent vascular access of 92:8 noted in our dialysis centre was unacceptably high compared to the internationally recommended 15:85.

## Background

Vascular access has continued to be the Achilles tendon of chronic maintenance haemodialysis [1,2]. There are two main types of vascular access: temporary haemoaccess via insertion of catheter into blood vessel (femoral vein subclavian vein, or internal jugular vein), and permanent haemoaccess (arterio-venous fistula and arterio-venous graft) [1-12]. Patients with end-stage renal disease on maintenance haemodialysis require creation of permanent haemoaccess like arterio-venous fistula (A-V F) early in the illness [3-5,12]. But for patients who present late, temporary haemoaccess may be used while awaiting the maturation of the A-VF [1,2].

Presently the double lumen internal jugular catheter is favoured for most cases requiring temporary haemoaccess [6-10]. Temporary haemoaccess is used predominantly in the management of acute renal failure and temporary plasma exchange [7]. However central venous catheter is sometimes indicated in the management of end-stage renal failure in patients with exhausted vascular access sites, non-suitable vessels, failed peritoneal dialysis or short life expectancy [7].

Alarmed by the rampancy of temporary vascular access with its attendant complications in our haemodialysis recipients for management of endstage renal disease, we set out to analyse the problems of vascular access in our new dialysis centre and to plan intervention.

## Methods

Dialysis records in the first one year of service covering January 2008 – January 2009 were retrieved and analysed. The following information were collated: age and sex of patients, total number of patients, indication for haemodialysis, number of haemodialysis sessions undergone by each patient, total number of haemodialysis sessions, vascular access utilized, vascular access related complications, and reasons for not using permanent vascular access in patients with end-stage renal disease utilizing temporary access. The data was manually analysed by percentage and proportion.

## Results

**Figure 1:**
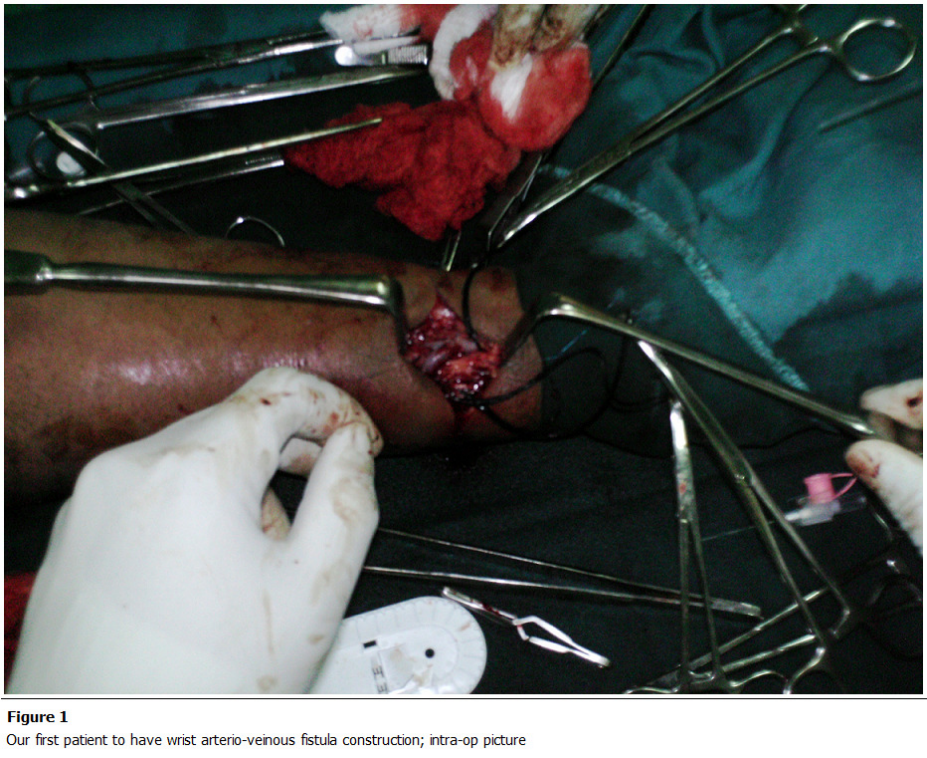
Our first patient to have wrist arterio-veinous fistula construction; intra-op picture

**Figure 2:**
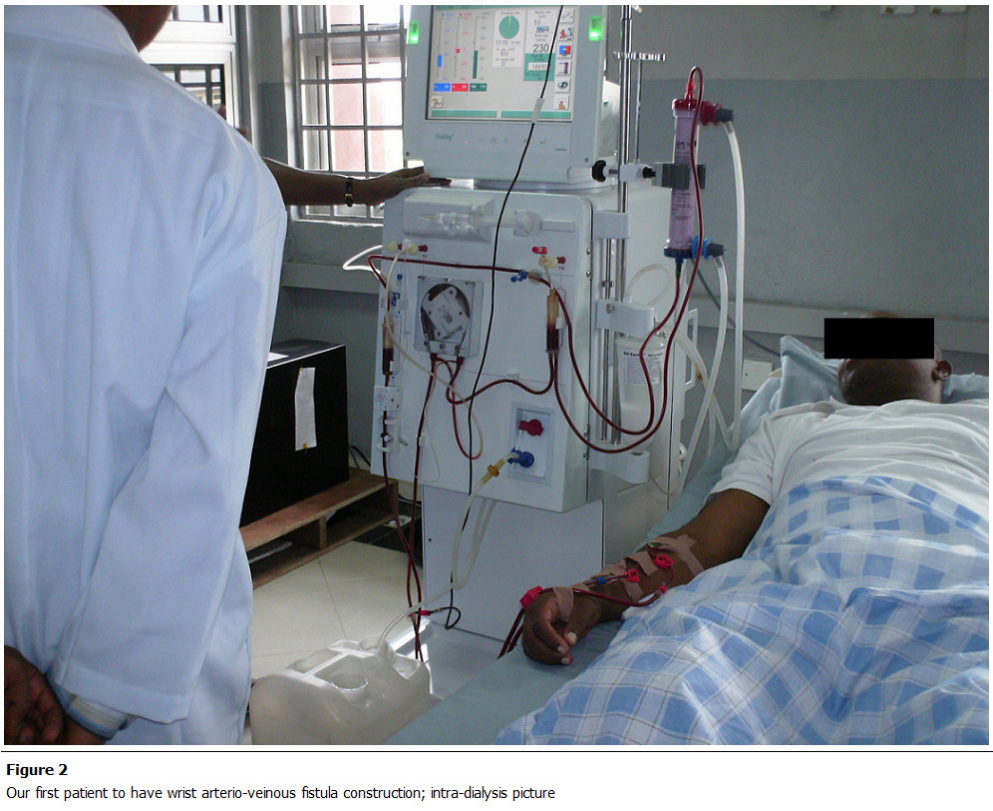
Our first patient to have wrist arterio-veinous fistula construction; intra-dialysis picture

**Figure 3:**
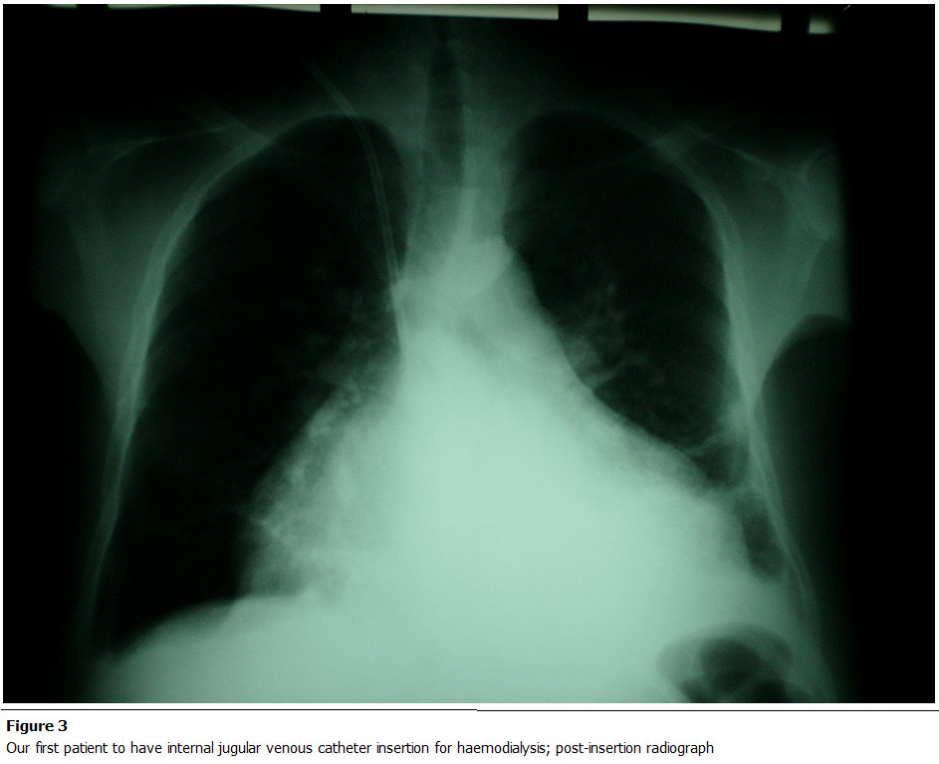
Our first patient to have internal jugular venous catheter insertion for haemodialysis; post- insertion radiograph

There were a total of 60 patients that underwent haemodialysis in our institution during the period under review. There were 38 males and 22 females giving male to female ratio of 1.7:1. Their ages ranged from 12-72 years with mean of 52 years. There were 3 patients with acute renal failure and 57 patients with end – stage renal disease. Only five (8.3%) of the patients had permanent vascular access while the remaining 55 (91.7%) of the patients had temporary vascular access (Table 1 and 2). There were total of 254 haemodialysis sessions undergone by 60 patients during the period under review, with 234 (92%) haemodialysis sessions undergone via temporary haemoaccess, while only 20 (8%) haemodialysis sessions were via permanent haemoaccess. The complications recorded included blockage/thrombosis of catheter in 30 cases, difficult cannulation in 10 cases, haematoma, poor flow in 7 cases each, kinked catheter in 4 cases, haemorrhage in 2 cases and infection in 1 case giving a complication rate of 24% (table 3). Figure 1, 2 and 3 show some procedures being conducted in the new dialisys center.

Reasons attributable to low level of utilization of permanent vascular access included non-referral (7.8%), late referral (11.5%), refusal by patients and or relatives (3.9%), unavailability of fund (30.8%), and unavailability of synthetic vascular graft (11.5%) (Table 4).

In the remaining 34.7% of end-stage renal disease patients who underwent haemodialysis via temporary vascular access no reason was given.

## Discussion

With the availability of dialysis, the lives of patients with end-stage renal disease have been greatly prolonged [13]. Vascular access capable of allowing the flow of >200ml/minute of blood greatly enhances haemodialysis [3]. Patients with aetiological risk factors for end-stage renal disease should be followed up for both clinical and biochemical indicators of deteriorating renal function. When it can be predicted that haemodialysis would be indicated in another one to two months, the patient should be counselled and referred to the vascular access surgeon for assessment and creation of permanent vascular access to enable maturation ahead so that the first haemodialysis can be done via a permanent vascular access [13]. It is only with the adoption of this kind of protocol that a nephrology unit can achieve the internationally recommended 15:85 temporally to permanent vascular access ratio for haemodialysis in end-stage renal disease patients [13]. This is also advantageous as a study has noted a 25 – 30% higher corrected mortality rate among end-stage renal disease patients using central venous catheter for maintenance haemodialysis than those using arterio-venous fistula [7]. In USA, about 17% of such haemodialysis are done via central venous catheter, while 83% are via arterio-venous fistula [7]. In Europe and Japan, the corresponding figures for haemodialysis via central venous catheter are significantly lower at 8% and 3% respectively [7].

Tables 1 and 2 show that all known varieties of both temporary and permanent types of vascular accesses were utilized for our patients during the study period except cuffed subclavian catheter, which however was subsequently used for a few haemodialysis patients. However the ratio of patients using temporary vascular access to those using permanent vascular access of 55:5 (92% versus 8%) portends suboptimal patient care. After critical analysis of vascular access for haemodialysis, Uldall concluded that Subclavian cannulation is no longer necessary or justified in patients with end-stage renal failure [14]. The same tables 1 and 2 further depict the percentage of haemodialysis through temporary vascular access in comparison with that through permanent vascular access of 92% versus 8%. This was significantly at variance with the internationally recommended 15% versus 85% [13]. It was also not surprising that the vascular access related complication rate was as high as 24% (table 3). Ninety-nine per cent of these complications occurred in temporary vascular accesses which therefore mean that a reduction in the proportion of temporary access would positively be correlated with a reduction in vascular access related complication rate.

The initial lessons learnt when the preliminary data was made public to the hospital community resulted in some attitudinal change with increase in the number of patients with end-stage renal disease referred early for creation of arterio-venous fistula. The impact of this attitudinal change currently is an increase in the proportion of haemodialysis done via permanent vascular access and a corresponding decrease in the proportion of haemodialysis done via temporary vascular access with its attendant complications (unpublished data).

Unavailability of fund on the part of the patients ranked highest among the reasons for non-utilization of permanent vascular access (Tables 4). This is a serious concern because the medical and surgical management of end-stage renal disease are expensive everywhere in the world. Currently our centre charges an equivalent of $US 600.00 per week for maintenance haemodialysis. Besides the scarcity of living kidney donors, the few kidney transplantation centres in Nigeria charge an equivalent of about $US 34,000.00 on the average for kidney transplant operation. Sadly too, the treatment of chronic renal disease is one of the exclusions in our country National Health Insurance Scheme (NHIS). Currently our vascular surgery unit is sourcing for grant for the procurement of prosthetic vascular graft for purpose of arterio-venous grafting in patients with nonsuitable autogenous vessels for arterio-venous fistula. When this succeeds, we will be making good progress toward better maintenance haemo- dialysis service in our centre.

## Conclusion

The ratio of temporary to permanent vascular access of 92:8 noted in our dialysis centre was unacceptably high compared to the 15:85 recommended internationally. Prompt referral of end stage renal disease patients for vascular access procedure was emphasized. Grant for synthetic vascular graft is being pursued.

## Competing interests

We hereby declare that there are no conflicts of interest in this study.

## Acknowledgements

We acknowledge the contribution of medical personnel who helped in the retrieval of the dialysis records of the patients included in this study.

## Authors’ Contributions

The two authors have jointly conceived and designed this study, collected data, analysed and interpreted the data; and also took part in drafting the article and revising it critically for important intellectual content; and finally approved this version to be published.

## Figures and Tables

**Table 1:** Distribution of patients utilizing temporary vascular accesses for haemodialysis in a new dialysis centre in Uyo State, Nigeria, January 2008 – January 2009

**Vascular access**	**Frequency (%)**	**Haemodialysis sessions (%)**
Right Femoral catheter	27 (45.0)	98 ( 38.6)
Left Femoral catheter	11 (18.3)	51 ( 20.1)
Right and left Femoral catheter	13 (21.7)	62 (24.4)
Right Jugular catheter	3 (5.0 )	18 (7.1)
Left Jugular catheter	1 (1.7)	4 (1.6)
**Total**		**234 (92)**

**Table 2:** Distribution of patients utilizing permanent vascular accesses for haemodialysis in a new dialysis centre in Uyo State, Nigeria, January 2008 – January 2009

**Vascular access**	**Frequency (%)**	**Haemodialysis sessions (%)**
Left Radiocephalic AVF	3 (5.0)	16 (6.3)
Right Radiocephalic AVF	1 (1.7)	3 (1.2)
Thigh AV Graft	1 (1.7)	1 (0.4)
**Total**	**5**	**20**

AVF: Arterio-veinous fistula, AV: Arterio-veinous

**Table 3:** Vascular access related complications recorded in a new dialysis centre in Uyo State, Nigeria, January 2008 – January 2009

Complication	Frequency (%)
Catheter Blockage/Thrombosis	30 (1.8)
Difficult Cannulation	10 (3.9)
Haematoma	7 (2.8)
Poor Flow	7 (2.8)
Kinked Catheter	4 (1.6)
Haemorrhage	2 (0.8)
Infection	1 (0.4)
**Total**	**61 (24.0)**

**Table 4:** Reasons for non-utilization of permanent vascular access in 52 patients on maintenance haemodialysis via temporary vascular accesses in a new dialysis centre in Uyo State, Nigeria, January 2008 – January 2009

**Variable**	**Number (%)**
Catheter Blockage/Thrombosis	30 (1.8)
Non referral	4 (7.8)
Late referral	6 (11.5)
Poor Flow	7 (2.8)
Refusal	2 (3.9)
Unavailability of fund	16 (30.8)
No reasons	18 (34.7)
**Total**	**52 (100)**
